# State of nano pesticides application in smallholder agriculture production systems: Human and environmental exposure risk perspectives

**DOI:** 10.1016/j.heliyon.2024.e39225

**Published:** 2024-10-10

**Authors:** Jones Ackson Kapeleka, Mwema Felix Mwema

**Affiliations:** a Tanzania Plant Health and Pesticides Authority (TPHPA), P.O. Box 3024, Arusha, Tanzania; b School of Materials, Energy, Water and Environmental Sciences, The Nelson Mandela African Institution of Science and Technology, P. O. Box 447, Arusha, Tanzania

**Keywords:** Nanoformulations, Nanoparticles, Nanotechnology, Pesticides exposure, Greener pesticides

## Abstract

Due to the intensive and widespread use of agrochemicals, especially pesticides, agriculture in the majority of the world is in dire need of practical improvements to fulfil the rising need for food while at the same time decreasing its associated health and environmental impact. Traditional methods, such as integrated pest control, have been used extensively and globally for decades to lessen the effects of intensive and extensive pesticide use, but they are insufficient. Safer pesticide alternatives, including biopesticides, to replace conventional pesticides have also been developed, but these efforts have not yet reached the necessary degree of operationalization and commercialization. In light of the challenges and trade-offs involved in using conventional pesticides, nanotechnology has sped up the development of nanopesticides, that are poisonous solely to specific pests and pathogens. The effectiveness of nano-agrochemicals has often demonstrated a median gain compared to traditional products of 20–30 %. The use of nanopesticides may enable more precise pest targeting, reduced pesticide dosage and decreased spray frequencies, allowing for a 10-fold reduction in pesticides dosage without sacrificing effectiveness. However, there are environmental concerns and potential for human exposure associated with the use of nanopesticides. This state-of-the-art review examines the most recent advances in science and the application of nanotechnology as a unique tool to address the serious negative effects of conventional pesticides. In addition to the health and environmental implications, policy and regulatory framework, and field application of nanopesticides in smallholder production systems are all part of the scientific review that is presented in this review.

## 1 Introduction

Pesticide use has globally become mandatory in many agricultural production systems. The use of chemical pesticides in an attempt to control pests and boost farm output is influenced by the influx of agricultural pests and diseases as well as increased pest resistance to pesticides [1]. In most developing countries, agricultural production is largely dependent on the use of conventional pesticides due to climatic conditions that favours the development and emergence of a wide range of pests and diseases [2].

The use of these agrochemicals has therefore continued to persist in the agricultural sectors for decades [3]. Huge volumes of pesticides are imported in developing countries mainly from Europe and Asia. The amount of export value for the top 10 African countries (all from sub-Saharan) had reached 85.9 % of the total export value to Africa from China in the recent past [4]. Organophosphates, carbamates, and pyrethroids constitute the most insecticides, herbicides, fungicides, and rodenticides used due to their effectiveness, ease of application, availability, and relatively low cost compared to other control methods [[5], [6], [7]]. But these chemical compounds are not only toxic to both humans and wildlife but also they are difficult to degrade under natural environmental conditions [2,8].

The escalation of the use of conventional pesticides in smallholder agriculture production systems elevates the risks of exposure. Non-selective pesticide use is putting some species, such as birds, insect pollinators, or natural enemies of pests, at risk [8]. Paradoxically, most farmers use these pesticides without proper knowledge of the health and environmental effects [9,10]. While other studies show that the PPE does not provide adequate protection from the pesticides [11] that are being sprayed, exposure effects may be minimized when personal protective equipment are effectively used. By using appropriate PPE, the health effects of pesticides can be reduced [12].

The use of eco-friendly approaches to pest and disease control such as nanotechnology, biotic and abiotic inducers, biocontrol agents, and other cultural methods can be used to effectively control crop pests and diseases [13,14]. In this regard, nanotechnology has emerged as one of significant scientific advancements in the context of agriculture's transformation as well as efforts to address several health and environmental issues brought by the intensive and widespread use of conventional pesticides [[15], [16], [17]].

Despite its enormous promise, nanotechnology's uses in developing nations are primarily restricted to the treatment of disease and the prevention of health problems rather than crop protection [18]. As a result, the use of nanotechnology in smallholder agriculture is still in its infancy, and there is little knowledge about how it will affect people, animals, and the environment [16]. Research on the effectiveness of nanoparticles in improving pesticide effectiveness, plant growth, physiological, yield, mitigation of pest resistance, and biotic and abiotic factors [14,[19], [20], [21], [22], [23]] has been conducted in different parts of the world, yet their applicability in smallholder farming had not reached commercialization in most developing and underdeveloped countries.

This state-of-the-art review offers a summary of the most recent advancements in the use of nanotechnology in the creation of nanopesticides as a potential substitute for traditional pesticides. It aims to compile empirical data from research on nanopesticides conducted globally that could be of interest to developing countries like Tanzania and the rest of the underdeveloped world. The key motivation for this review is to derive scientific development regarding the applicability of nanotechnology in smallholder agriculture production and to assess readiness, political will, and the adoptability of nanopesticides in developing nations where the use of highly hazardous pesticides is alarming. Additionally, it presents summative instances of the inherent advantages of nanotechnology to smallholder agriculture production systems and analyzes the current status of research and field application of nanopesticides. An evaluation of human and environmental exposure risk and other potential negative impacts of nanopesticides pesticides is presented.

## 2 Materials and methods

The review covered 107 published research papers including reviews and research articles. The Preferred Reporting Items for Systematic Reviews and Meta-analysis guidelines (PRISMA Statement) was employed [24]. Google Scholar, Agora, Hinari, and PubMed were used to collect relevant papers from science databases. A total of 887 were collected. After screening for duplicates, 421 papers were obtained. Only papers with the following search words: pesticides, nanopesticides, nanoformulations, metal and metal oxide nanoparticles, pesticides exposure, agriculture, organic and inorganic nanopesticdes, nanotoxicity, environmental exposure, pesticides use; in their title, abstracts and keywords fulfilled the inclusion criteria and hence included. The search was limited to papers from 2000 to 2024 only whereby 218 papers were collected. Topic-based search was further applied by combining two or more search words with ‘AND’ and hence 107 papers met these criteria and were included in the list of papers that were reviewed. No geographical restriction on review coverage was applied, yet, only papers published in English were considered for review.

## 3 Nanotechnology and its application in pesticide formulation

Nano is the term that refers to materials having a nano-meterrange (1–100 nm) in at least one of its dimensions [25]. Nanopesticides are therefore plant protection products that use nanotechnology to increase a pesticide's effectiveness while lowering its negative effects on the environment and human health. They are simply small engineered structures that deliver pesticidal properties or formulation of the active ingredient of pesticide in nano form [26]. Nanopesticides can be combined with polymers, surfactants, and metal nanoparticles to form pesticide formulations with innovative pesticidal characteristics that fall within the nanometer size range [6,8,15,16,[27], [28], [29]].

The primary properties of nanopesticides are their small particle sizes and high surface area-to-volume ratios. This speeds up chemical reactions in the biological domain more accurately and effectively, thereby improving pesticide deposition and lengthening the sustained pesticide on crop leaf release period [29]. When compared to conventional pesticides, nanopesticides are applied less frequently and in smaller amounts, which helps conserve energy and water use. This might benefit smallholder farmers who rely on renting spray pumps and hiring spray men because it lowers the cost of pesticide application [30].

The chemical nature of the nanocarrier can also be used to classify nanoformulations. Among these are formulations based on lipids, organic polymers, layered double hydroxides, nanoscale metals and metal oxides, clay-based nanomaterials, and silica nanoparticles [8]. Formulations for organic and/or inorganic nanopesticides comprise substances that are created by mixing or combining nanomaterials of both an organic and/or inorganic nature. These substances boost the benefits of shielding/protecting the active components from premature deterioration and aid in the controlled release of the active ingredients [31]. Proteins, lipids, and polymers are the primary ingredients in the production of organic nanopesticides [6,32,33].

Inorganic chemicals with pesticidal capabilities, such as metals like silver (Ag) and copper (Cu), metal oxides (including Al_2_O_3,_ TiO_2_, SiO_2,_ ZnO), carbon nanotubes, or their mixtures, are included in the inorganic pesticides nanoformulations [34]. These inorganic nanoparticles, which include silver, gold, copper, copper oxide, titanium dioxide, and zinc oxide, exhibit strong antibacterial properties [35].

Pesticide-loaded nanocapsules are typically made in a variety of methods. Some of these methods include emulsion-solvent/evaporation and polyelectrolyte complexation [8,16]. Emulsions are the most popular lipid-based formulations utilized in agricultural applications globally [16]. Water, water-immiscible oils, surfactants, co-surfactants, and pesticides make up their intricate complex mixture. In the course of polymerization, appropriate monomers can be used to create polymeric nanocapsules [8]. Hexaconazole, for instance, can be transformed into nanohexaconazole by being enclosed in polyethylene glycol-400 [36]. It should be emphasized that nanocapsules may be created to release their active components under extremely precise environmental circumstances, such as in an insect's stomach [6].

## 4 Classification of nanopesticides

The nanopesticides are classified into three main categories based on their chemical nature; that is organic (nanocarriers made of polymers, chitosan clays, etc) and inorganic nanomaterials (metal-based such as silver (Ag), copper (Cu), aluminium (Al), and titanium (Ti) as well as a combination of organic and inorganic nanomaterials. Active ingredients and nanocarriers, which stabilize the active substances in the nanosize range, make up the two essential components of nanoformulations [6,37]. They come in various forms, each designed to enhance pest control and minimize environmental impact. The most synthesized types of nanopesticides include nanocapsules, nanoemulsions, nanogels, nanospheres, and metal and metal oxide nanoparticles.

Nanocapsulated formulations involve tiny capsules that encapsulate pesticide molecules. They protect the active ingredients, control release, and improve efficacy [[38], [39], [40], [41]]. Nanoemulsions are oil-in-water or water-in-oil emulsions containing nano-sized pesticide droplets. They enhance pesticide stability, solubility, and penetration into plant tissues [[42], [43], [44]], while nanogels are gel-like structures at the nanoscale that can carry pesticides. They offer controlled release and targeted delivery [45,46]. Nanospheres are spherical nanoparticles loaded with pesticides. They improve tissue permeation and reduce volatilization [6,47]. Metal and metal oxide nanoparticles (such as silver, zinc oxide, or copper hydroxide) can be loaded with pesticides. They provide antimicrobial properties and targeted action and can be used directly as nano pesticides without carriers [21,48,49]. The other form of nanopesticides is inorganic porous nanoparticles. These versatile nanoparticles have a wide range of mechanical and physicochemical properties. They can serve as promising nanocarriers for delivering pesticides in agriculture. Pesticides can be coated onto nanostructured materials like clay nanoparticles or carbon nanotubes. These materials enhance adhesion and slow down pesticide degradation [50,51].

**Fig. 1:**
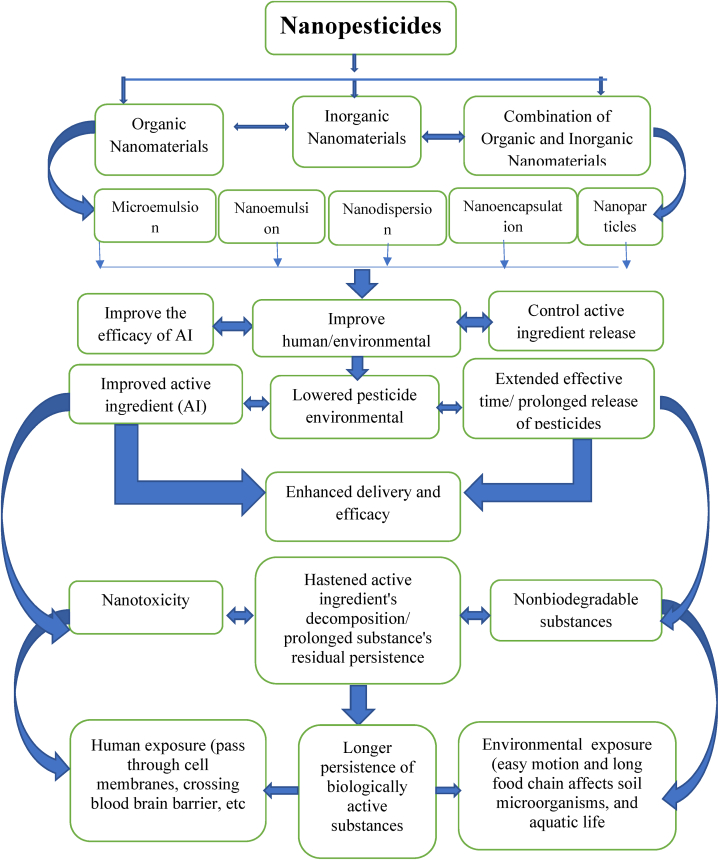
Classification of Nanopesticides and their health and environmental implications.

Generally, capsules, fibers, emulsions, gels, and nanoparticles can be employed as carriers for the real active ingredient. Nanoparticles (such as copper) can act alone in a formulation as nanopesticides [6,16,52]. Moreover, nanopesticides containing pristine engineered nanoparticles such as metals, metal oxides, and nanoclays have been developed [15,32] in microemulsion, nanoemulsion, nanodispersion and nanoencapsulation formulations [53]. By nanostructuring the pesticide active ingredient, the residual effects of the majority of pesticides can be efficiently controlled. Engineering the nano delivery systems enables the nanopesticides to overcome environmental and biological barriers that prevent the counterpart conventional pesticides from reaching the target [8]. The classification of nanopesticides and resulting health and environmental implications are summarized in Fig. 1.

## 5 Efficacy of nanopesticide formulations over conventional pesticides

Nanotechnology-developed formulations have proved some advantages over conventional pesticides as they remain active and stable under a variety of environmental conditions [53]. They have the potential to reduce the usage of conventional pesticides and improve soil health while being helpful for sustainable management and effective control of a variety of pests [20,30,49]. Nanoparticles can be effective in overcoming biotic and abiotic stresses through increased nutrient uptake under water stress conditions, modulating plant enzyme activities, and carbohydrate metabolism [49,54,55]. They are efficient in improving the biochemical traits of the plant, resulting in enhancements in plant tolerance, improved growth, productivity, and quality traits [55]. Improved active ingredient (AI) stability, an extended effective time, and lowered pesticide environmental burden are anticipated to increase pesticide efficacy [15,36,37,52,56].

The use of nanotechnology in agriculture enables targeted and regulated administration of pesticide active ingredients preventing their loss by physical, chemical, and microbial processes, enhancing stability, and decreasing volatility hence reducing risks associated with environmental impacts, toxicity, and insect resistance of conventional pesticides. The delivery mechanism for nanopesticides enhances their resistance to deterioration when exposed to environmental conditions [6,8,26,57,58]. By limiting early degradation and boosting bioavailability, gradual and controlled release of pesticides increases their effectiveness by increasing uptake by the target insect. Nano-formulation can also increase the solubility and dispersion of lipophilic pesticides in water while binding, absorbing, and transporting compounds, resulting in a prolonged release of pesticides while reducing the adverse effects of the pesticides on non-target organisms and the environment [33]. This drastically lowers the requirement for frequent pesticide application and the quantity of pesticides used [16,29,59].

Moreover, pesticides' nanoformulation effectively controls the stability, sensitivity, and photodegradation of their active ingredients [36]. The effectiveness of nano-agrochemicals has often demonstrated a median gain compared to traditional products of 20–30 % [[60], [61], [62]]. Several nanopesticides have been developed and have shown to be highly effective against their intended pests in comparison to commercial pesticides.

Sulphur and hexaconazole nanopesticides had been found to have better activity against mites and fungi (*Rhizoctonia solani*), compared to its WDP formulation. This nanosulphur was ten times more efficient in controlling mites while nanohexaconazole was five times more effective at controlling fungi [32,36]. The effectiveness of methomyl-loaded nanocapsules [41] was superior to the original, with a control efficacy of 100 % over 7 days for controlling armyworm. Similarly, powdery mildew might be effectively controlled with nano fungicide compared to its original formulation [36,56].

Nanopesticides can be instrumental in the management of exposure. The exposure effects of the parent active components are lessened by the nanoformulation of pesticides. For instance, nanoencapsulating paraquat considerably reduced the genotoxic effects of paraquat [62,63]. When compared to traditional paraquat herbicide, nanoparaquat-treated samples showed less chromosome damage, suggesting that nanoencapsulation is a potential strategy for reducing the genetic harm brought on by bulk paraquat [62,64,65]. Thus, nanoencapsulation has the benefit of safer handling and more effective pesticide usage with less exposure effects.

The effectiveness of metal and metal oxide nanoparticles against several agricultural diseases and pests has been widely studied. Due to their chemical composition, metal nanoparticles have a harmful effect on plants, which is linked to chemical toxicity [6]. Traditional organosynthetic fungicides can be replaced with nanosilver. This is due to the excellent action of silver nanoparticles (10–50 nm) against *Rhizoctonia solani, Botrytis cinerea, Sclerotinia sclerotiorum, Alternaria alternata, Curvularia lunata*, and *Macrophomina phaseolina* [31,56]. Furthermore, silver has greater antibacterial activity than other metals like mercury, copper, lead, chromium, and tin. Because the silver nanoparticles in nano silver colloids are highly disseminated and stabilized, they are more sticky on the cell surfaces of bacteria and fungi, making them excellent fungicides [6,58]. ZnO nanoparticles, on the other hand, offer a promising substitute treatment for managing plant diseases and pests in a far more sustainable way as they are less harmful to plants than silver nanoparticles [32].

Studies have shown that *Fusarium graminearum*, which causes catastrophic disease of wheat and cereal grains, can be treated with pure chitosan (CHI) nanogels loaded with Cu(II) ions [46]. Furthermore, copper nanoparticles that were created and coated with Cetyl Trimethylammonium Bromide (CTAB) significantly inhibited the growth of plant pathogenic fungi such as *Phoma destructiva*, *Curvularia lunata*, *Alternaria alternative*, and *Fusarium oxysporum* [66]. Pesticides zineb and mancozeb were encapsulated on multiwall carbon nanotubes and grafted with poly(citric acid), which had a more harmful effect on *Alternaria alternative* fungi [67]. Moreover, copper nanoparticles were discovered to inhibit the growth of bacterial blight (*Xanthomonas axonopodis* pv. *punicae*) at concentrations >10,000 factor lower than those of copper oxychloride [68]. The investigated fungi and bacteria, such as *Aspergillus nidulans*, *Trichaptum biforme*, *Penicillium italicum*, *Escherichia coli*, *Citrobacter freundii*, and *Fusarium oxysporum*, were completely inhibited by smaller-sized spherical and quasi-spherical silver nanoparticles [69]. Nanoencapsulated imidacloprid (IMI) was found to be more successful at controlling grasshoppers than free imidacloprid when it was added to alginate (ALG) nanocapsules using the double emulsion method [62].

The use of nanoherbicides for weed control is viewed as an economically sound substitute for traditional herbicides [61]. Tests on target plant (*Brassica* sp.) and nontarget maize (*Zea mays*) using a nanoparticle formulation containing the herbicide atrazine (1 mg/mL) showed that nanoencapsulation did not change the herbicide's action mechanism and that it was exclusively effective against the target organism. Likewise, the 2,4-D nanosized rice husk nanoformulation was found to exhibit better herbicidal activity against the tested target plant (Brassica sp.) compared to the conventional 2,4-D, along with increased sustained release of 2,4-D in both water and soil [57].

As compared to a commercial atrazine formulation, the polymeric nanocapsules of atrazine not only allowed for a slow release of the herbicide but also improved the herbicidal action, allowing for a 10-fold reduction in atrazine dosage without sacrificing effectiveness [34], precipitating no any phytotoxicity to the non-target plant as it does to the target plant [64,70,71]. Likewise, paraquat-loaded Chitosan-Sodium Tripolyphosphate nanoparticle was found to be more successful in the initial eradication of weeds, with delayed release from nanoparticles providing subsequent control than traditional paraquat in maize [64]. Since most farmers mix liquid fertilizers and pesticides, nanopesticides can be designed to improve tank-mix compatibility, enabling the development of a homogeneous and adequately stable solution with liquid fertilizers and/or other pesticides for combined application [60].

Leaching and runoff of pesticides can be decreased, and the resulting residual effects of conventional pesticides can also be reduced, because of nanoencapsulation. The delivery systems at the nanoscale boost efficiency by adhering firmly to plant surfaces and cutting back on pesticide usage by preventing runoff into the environment [58]. However, nanopescticides may also result in the production of new soil and water contaminants due to longer persistence, enhanced transport, and mobility as well as higher toxicity [72].

The nanoformulated pesticides were substantially less hazardous in environmental toxicology trials. Nanohexaconazole had no significant negative effects on soil enzyme activities, nitrogen-fixing bacteria, blue-green algae, or total soil microbial count [36]. Furthermore, chitosan-encapsulated paraquat did not alter soil macro- and micronutrients, enzymes, soil microflora, seedling emergence, or plant growth characteristics in *Vigna mungo* plants when they were combined in a nanoformulation [73].

Nanoparticles can also be used to manage soil contamination caused by conventional pesticides. Under regulated environmental conditions, the application of silver nanoparticles (AgNPs) stabilized with carboxymethyl cellulose (CMC) was found to breakdown 88 % of atrazine, which could be a useful remedial measure of bioremediation, eliminating the leftover problem related to atrazine [70]. It has been suggested that atrazine nanoencapsulation can decrease the genotoxicity of atrazine and the risk associated with occupational and environmental exposure [71]. Likewise, when compared to the technical 2,4-D, the nanoformulation of 2,4-D showed the effectiveness of nano-sorbent in controlling the herbicide run-off to the closest water bodies [57].

Research advancement on nanoformulated pesticides has therefore shown that nanopesticides play a significant role in production cost reduction, boosting agricultural product yield, and achieving precision agriculture [59]. The effective usage rate of pesticides is increased and environmental loss is minimized by the stable and controlled release of pesticide using nanoparticles having a high affinity for crop leaves [74]. Given this, it is possible to significantly reduce pesticide losses in smallholder agriculture. Hence, using nanopesticides may enable more precise pest targeting, usage of smaller amounts of a pesticide, and reducing the frequency of spraying, all of which may improve human and environmental safety while lowering the high costs associated with pest control (Table 1).

**Table 1:** Efficacy of selected nanopesticide formulations.

Nanopesticides/particles	Target/test pest/pathogen	Efficiency	Reference
Silica nanoparticle	Cotton leaf worm (*Spodoptera littoralis)*	High mortality rate (96 %) compared to commercial silica	[75]
Silver nanoparticles	*Spodoptera litura*	High mortality rate (up to 86 %)	[76]
Silver nanoparticles	Plant pathogenic fungi like *Fusarium* sp. and *Phoma* sp	Effectiveness against plant pathogenic fungi	[77]
Calcium carbonate nanoparticles	California red scale (*Aonidiella auranti*) and Oriental fruit flies (*Bactrocera dorsalis*)	High detrimental effect	[78]
Silver nanoparticles–chitosan encapsulated paraquat	Invasive weed specie *Eichhornia crassipes*	Improved herbicidal activity was observed against *E. Crassipes*	[73]
Silver nanoparticles prepared using algae *Chroococcus dispersus* and *Chlorella vulgaris*	Plant pathogenic bacteria and fungi	Antibacterial activity of AgNPs was four to five times higher than chemical antibiotics	[79]
Atrazine and simazine-containing solid lipid nanoparticles	Treatment of the target species *Raphanus raphanistrum* before and after emergence	Atrazine's herbicidal efficacy was increased through nanoencapsulation compared to commercial atrazine formulation	[34]
Copper nanoparticles with a cetyltrimethylammonium bromide coating as a capping agent	Plant pathogenic fungi *C. Lunata*, *A. Alternata*, *F. Oxysporum*, and *Phoma destructiva*	Superior to the widely used fungicide Bavistin against fungi that cause plant disease	[66]
Copper-chitosan nanoparticles	Plant pathogenic fungi *A. Alternata*, *M. Phaseolina*, and *R. Solani*	Showed 89.5 %, 63.0 %, and 60.1 % growth inhibition of *A. Alternata*, *M. Phaseolina*, and *R. Solani*	[80]
	Phy-topathogenic fungus *F. graminearum.*	Chitosan nanogels enhanced the combined antimicrobial activity	[46]
Abamectin poly(lactic acid) (Abam-PLA) nanoparticles		Exhibited better photostability and continuous release behaviour compared to active abamectin.	[81]
Paraquat-loaded chitosan/tripolyphosphate nanoparticles	Weeds in maize	Paraquat had a 62.6 ±0.7 % efficiency interaction with the nanoparticles.	[64]
Luteolin tetraphosphate derived silver nanoparticles and Gold nanoparticles	Plant-based Bacteria and fungi	In comparison to larger size nanoparticles, smaller silver nanoparticles demonstrated good growth inhibition of *Aspergillus nidulans*.	[69]
Nano-silver particles produced by Micro Algae	Plant pathogenic bacteria and plant pathogenic Fungi	Nanosilver showed high activity against the examined fungi 9 times higher than the activity of the generic antibiotics	[79]
Palladium nanoparticles	*Colletotrichum gloeosporioides* and *Fusarium oxysporum*	Palladium nanoparticles played a critical role in their antifungal Activity	[82]

## 6 Nanotechnology application in agriculture and challenges for adoption

Research on nanopesticide has gained high-speed recognition and interest mainly in agrochemical and other research laboratories. Nonetheless, these areas of exploration, advancement, and applicability are yet to reach public awareness and state authorities, as a result, nanoformulated pesticide products are not readily available in the market [72]. The development of pesticide nanoformulations, such as coating, encapsulation, and nanoemulsions, is ongoing, and it is likely a long way before these nanoproducts are used in farms in emerging and impoverished nations [27]. Paradoxically, limited research on the creation of nanopesticides for agricultural production has been reported in Africa [75,79] and there have been no clear reports of any field applications of the same. Smallholder farmers in these nations are therefore long way to benefit from advances in nanotechnology, which can boost output while reducing exposure to hazardous conventional pesticides. Certain nanoparticles' resilience and reactivity pose environmental issues, and the lack of information on how exposure to engineered nanoparticles affects the environment could limit their acceptability [30].

The advantages of nanopesticides use in agriculture against conventional pesticides have triggered research and the diffusion of nanopesticides in various countries including the United States, Canada, Asia, Australia, and Europe of recent. Worldwide, the USA receives the most money for nanotechnology research, followed by China, Germany, and Japan [74]. However, the majority of research on nano-assisted agriculture has focused on controlling tests (under controlled conditions), and there is little information on their application in the field [30]. Paradoxically, no evidence of studies was found in Sub-Saharan Africa where intensive and extensive pesticide use had been reported.

Moreover, specific legislation and guidelines for their use and commercialization are not readily available in every country [83]. For the nanopesticides to be widely adopted by the farming community and gain commercial production stage, the policy and regulatory environment of the recipient nation must support this advancement in nanotechnology. The possibility that nanomaterials could have harmful impacts on both people and the environment therefore becomes a major reason for concern. This is because engineering a chemical substance using nanotechnology can be compared to producing a new chemical substance [16], hence the existing chemical development guidelines in developing as well as developed nations may probably not fit the engineered nanopesticides due to differences in physicochemical properties between nanopesticides and their conventional analogues [52].

The majority of nations have not shown much support for the approval and authorization process for plant protection products and nanosized active chemicals. Few developed nations such as the EU had ratified regulations to govern nanopesticides. Regulation (EC) No. 1107/2009 on the EU governs the registration, assessment, authorization, and restriction of chemicals in EU member states, and also controls the use, characterization, and application of nanopesticides. Yet, there is scarcely any explicit regulation on nanopesticides in developing nations [84].

The effectiveness, physicochemical properties, behaviour, environmental fate, transformation, and toxicity of nanoformulated pesticides must be thoroughly assessed based on defined testing criteria before registration and commercial manufacturing [52]. The registration and commercialization of nanoagrochemicals should take into account both efficacy and safety concerns [85]. However, it is important to note that the environmental dangers of nanopesticides cannot be reasonably and completely assessed using the current methods for regular pesticides [59].

Although there are many theories about how conventional pesticides can be enhanced by using nanomaterials, there are currently very few nanopesticide products available. This is presumed to have resulted from inadequate public funding, a tardy adoption of this invention by agricultural research institutions, the low profitability of the agricultural sector, or a lack of widespread adoption of technology [85]. To prevent this revolutionary technology from encountering the same concerns and bottlenecks as genetically engineered crops, nanomaterials should be thoroughly evaluated and science-based evidence produced [36], and to properly evaluate the relative merits of a nanoformulation before it is registered or commercialized. It must be compared to the appropriate conventional formulation [85].

Nanopesticides are reportedly effective at controlling crop pests and disease-causing microbes in agriculture [14,20,49,86], yet, research on their use is still in its infancy. It is primarily laboratory-based, with very few field applications, and there is little information on their use by smallholder farmers. Its commercialization in Sub-Saharan Africa, developing and underdeveloped nations across the globe is scantly documented, hence limiting its applicability in smallholder production systems. Registration process and guidelines for nanopesticides must be established especially in countries where there is none [36]. A thorough assessment of a product's physicochemical characteristics, environmental destiny, toxicity, and ecotoxicology, including exposure data and dose response, is necessary for its commercialization and effective use [16].

## 7 Toxicity of nanopesticides and negative consequences

Toxic pesticides may have fewer detrimental effects on the environment if they are nanoencapsulated. However, to prevent environmental toxicity and unintended consequences on species, questions about nanoparticles need to be addressed for a better knowledge of these materials [63]. This is because the behaviour of any pesticide released to the environment and its end results are vital in undertaking the novelty of technology to reduce exposure effects. Undeniably the use of nanopesticides had been associated with negative and positive effects on the soil microbial communities [37].

The effects of nanopesticides in the environment are too complex to be clearly understood due to complex interactions about the developmental stages and mode of delivery of specific nanoparticles in the target or non-target living organism [37]. However, several studies [74,76,85,87,88] have shown that nanoformulations have toxicological consequences.

Due to the dangers to human and animal health as well as the environment, the use of nanopesticides by all parties involved, notably smallholder farmers who are the primary consumers, requires extensive understanding [16]. Public concerns about nanopesticides in terms of health, safety, possible toxicity to non-target organisms, transportation, bioaccumulation, and interactions with other pollutants in the environment [83] must be clearly understood. Contrarily, research on the potential negative effects of nanopesticides on the environment is still far behind the development of their use and exposure assessment [59]. Yet, a pesticide's fate and behavior both during and after application in the receiving environment are crucial factors that determine how it may affect ecosystems and human health [60].

Nanoparticles may be harmful to both aquatic and terrestrial organisms, as evidenced when pyrethrum extract nanoparticles caused genotoxic responses in DNA damage in tadpoles [63]. When exposed to nanoparticles containing the herbicides atrazine and simazine, roundworm *Caenorhabditis elegans* died at a higher rate than untreated worms [88]. Nanomaterials can change physically, chemically, or biologically when they come into contact with soil, depending on their makeup and how they interact with different organic and inorganic soil components [30]. The content, size, and concentration of the nanoparticles are the key determinants of the level of nanotoxicity in soil, plants, and water. Enhancing the active ingredient's solubility could also hasten the active ingredient's decomposition by soil microorganisms and increase its mobility [28].

Nanopesticides may result in both human and environmental exposure. CuO nanoparticles induced DNA damage in agricultural and grassland plants demonstrated phytotoxic effects, and limited root growth [87]. Although nanoformulations can change how agrochemicals behave in the environment, modifications may not always result in a decrease in the environmental impact Nanoformulations may affect the fate of pesticide-active components more than commercial formulations [60].

Damage to membranes, the production of reactive oxygen species, and genotoxicity are the most typical pathways for the harmful effects of nanoparticles. Nonbiodegradable substances have a higher risk of toxicity since they can accumulate in the body, stay there for an extended period of time, and stimulate the immune system [6]. Typically, nanoparticles smaller than 50 nm have a negative impact on human health, and possible routes of exposure include ingestion, dermal contact, and inhalation. Nanoparticles can also have harmful effects on soils, humans, plants, animals, and aquatic life due to their easy motion and long food chain [74].

A variety of soil microorganisms can be negatively impacted by nanoparticles, particularly those made of metals, metal oxides, zeolites, and clays, due to their physical impact on membranes, formation of ROS, and production of metal ions [89]. The presence of engineered nanomaterials in human tissues may have negative effects such as developmental abnormalities, infertility, disruption of brain and muscle activity, stress, and endocrine-related problems [90].

Earthworms and soil bacteria may have decreased development, fertility, survival, and higher mortality as a result of nanoparticles such as Cu(OH)_2_, Ag, TiO_2_, ZnO, CeO_2,_ and Fe_3_O_4_ [74,91]. An increase in the solubility of a nanopesticide can increase the active ingredient's mobility in the environment, potentially contaminating water sources. Moreover, the protection of a pesticide against early degradation might result in a longer persistence of biologically active substances, which may be detrimental to organisms other than the target [8,16,29]. The small size of several inorganic nanopesticides has also been linked to carcinogenic effects, which has hampered their commercialization in the European Union [31]. The toxic effects on selected nanopesticides are summerized in Table 2.

**Table 2:** Toxicity of nanopesticide.

Nanopesticides/particles	Target organ/media	Toxic effect	Reference
Zinc Oxide NPs (ZnONPs)	Cellular level (cytotoxicity), pregnant females and their fetuses	Damaging of genetic materials such as DNA strand breakage, crosslinking of DNA and genetic mutation,	[92,93]
Gold Nanoparticles AuNPs	Cell level, crustacean Daphnia magna	DNA damage, decrease in the hematocrit, and red blood cell count, mortality of parental females, impaired development and decreased reproductive capacity	[[94], [95], [96]]
Silver nanoparticles (AgPNs)	Brain cells, Reproductive cells	enhanced synthesis of ROS, cause changes in sperm morphology and decrease the number decrease in sperm count and viability of sperm	[93,97]
Copper based nanopesticides (Cu(OH)_2_)	Spinach plants cells, early life stages of zebrafish (*Danio rerio*), lung cell lines	Reduced antioxidant molecules including as ascorbic acid, alfa-tocopherol, threonic acid, 4-hydroxybutyric acid, ferulic acid, and total phenolic compounds, induce developmental toxicity with malformations in embryos/larvae, induction of reactive oxygen species (ROS) and genotoxic damage	[[98], [99], [100]]
Au NPs, Ag NPs, cobalt (Co) NPs and Si NPs	*D. melanogaster*	Somatic mutation, gene mutation, toxicity, impaired fertility and longevity,	[101]
Nanosized TiO_2_	Male reproductive organs, Germ cells	marked changes in body weight and the relative weights of the testis and accessory male sex organs, cause morphological changes in follicles and may lead to a reduced number of mature oocytes, direct DNA damage (chromosomic aberrations, mutation assays in mammals, sister chromatid exchange, etc)	[93,100]
Carbon nanotubes	Plant cells	Formation of reactive oxygen species (ROS) resulting in cell death and decreased soil microbial population	[102]

## 8 Discussion

Studies on nanopesticide formulations have demonstrated high levels of efficacy against target pests, significant reduction of the dosage, application frequency, and toxicity to non-target organisms, as well as residual and colloidal effects in the environment [20]. Increased pesticidal effects on target pests provide a promising mechanistic approach for the management of pesticide exposure in smallholder farming systems. The use of nanotechnology in the development of novel nanopesticides therefore, has the potential to lower the health and environmental exposure risks associated with conventional pesticides [103]. Nonetheless, for the novel nanotechnology to be effectively applied in smallholder agriculture, an evidence-based approach to their management has to be developed. Likewise, technology transfer packages must accompany research during their introduction into the farming system.

The application of nanotechnology in agriculture enables targeted delivery of active ingredients accompanied by controlled release of the active ingredient [6]. This mitigates the problems associated with conventional pesticide application methods such as toxicity, harmful environmental impacts and fewer efficiency issues by increasing the overall efficiency of pesticides [8,57]. Nanoformulation can increase solubility and dispersion of lipophilic pesticides in water while binding, absorbing, and transporting compounds, resulting in sustained release of pesticides [6,8]. Formulation of the active ingredients into nanopesticide formulations using nanomaterials as active pesticide agents or using them as nanocarriers for their delivery has been found to improve the efficacy while minimizing the negative effects of the pesticides on the non-target organisms and the environment [104].

Because they can be utilized as "smart delivery systems" for the release of pesticides in a timely yet controlled manner, during a chosen time frame, nanotechnology is anticipated to provide a unique platform to establish a dynamic balance between agricultural productivity and environmental sustainability [29]. Due to their major benefits, such as high efficacy at lower doses, easier pest control, fewer volatilization, and higher bioavailability [65], the success of nanopesticides will depend on the integration of their usage with traditional plant protection products [70].

Some studies have revealed the toxicological effects of nanoformulations [60,76,87,88,105] posing a serious concern in developing nations already overwhelmed by the toxicological effects of conventional pesticides. Excessive usage of nanopesticides may unintentionally release engineered nanomaterials into the environment, which is a major global problem that needs to be addressed. Nanopesticide products will therefore need to provide a clear advantage to farmers in terms of costs and benefits since conversational agrochemicals are already pricey [85]. Nevertheless, there is confidence that nanopesticides will provide new, advanced nano-based formulations that are stable and active in the spray environment, penetrate pests, can be delivered to the target pests/area, reduce pest/pathogen resistance, and remain safe for plants and mammals in addition to addressing the major limitations of current pest control strategies [33].

In order to produce nanoparticles, it is necessary to explore the use of microorganisms such as bacteria, fungi, and yeast as well as plants for the synthesis of metal nanoparticles. These organisms have the advantages of being non-toxic, reproducible in production, easy scaling-up, and well-defined morphology [106]. As an affordable alternative to traditional physical and chemical methods of nanoparticle manufacturing, this biological strategy appears to be promising [35].

Many gaps need to be answered for a better understanding of nanomaterials to avoid environmental contamination and undesired side effects on species [63]. However, nanopesticides are deemed environmentally friendly by manipulation of the highly toxic solvent and surfactants. In their study [82], synthesized clean, non-toxic, biocompatible, efficient, and environmentally friendly palladium nanoparticles which showed the highest antifungal activity for tested fungi. Likewise, the encapsulation of toxic pesticides in the polymers makes them less toxic remaining available to the target organisms for longer duration, hence minimizing significantly human and environmental exposure to pesticides.

Engineered nanomaterials often undergo chemical modifications based on dispersion and agglomeration over time. However, the concentration of the nanoparticles as well as several environmental factors like pH, and ionic strength of the medium play important roles in it. Thus, documentation of physicochemical characterization of nanopesticides at different levels of the environmental life cycle and detailed analysis of fate and effect studies are mandatory for the betterment of agricultural sustainable practices [3].

Moreover, nanopesticides' environmental fate and behavior are presumed to differ from those of conventional formulations, necessitating the adoption of new or enhanced risk assessment techniques when integrating them into IPM programs [16]. Extensive research is required to develop nanopesticides for commercial use and large-scale manufacture [61]. Efforts must be made to manufacture nanoparticles that are non-toxic to microorganisms and to establish soil management strategies that can inactivate nanoparticles and lessen their negative impacts on microorganisms [89]. More resources and research should be directed toward expanding the research and efficacy tests of developed nanoformulations in the ecological conditions of Sub-Saharan Africa and the underdeveloped world. Additionally, it is crucial to conduct mechanistic studies on the handling and application techniques of nanopesticides in underdeveloped and developing nations across different farming typologies. In this context, the projected increased activity of nanopesticides may lead to new concerns as well as new benefits for the welfare of people and the environment. A formulation's placement on the market should be determined by more than just a size threshold; rather, it should be based on a scientific evaluation of the risks and benefits associated with chemical products as well as the formulation's overall environmental performance [107].

## 9 Conclusion

The application of pesticide nanoformulations has shown improved effectiveness, lowered pesticide use, decreased pesticide residue in food, decreased danger of environmental contamination, fewer non-target impacts, improved economy, and increased safety for farmers or pesticide applicators. Nonetheless, nanopesticides have been linked to risks for the environment and human exposure due to their small size, enhanced solubility, and persistent release of the active ingredient over an extended period. The dangers associated with the use, adaptation, and commercialization of nanosized pesticides are increased by the relatively limited regulatory capability to regulate and reduce hazards and environmental aspects of nanopesticides. Lack of information and knowledge on nanopesticide handling and application procedures may reduce the likelihood of their adoption in smallholder agriculture production systems. Moreover, regulatory frameworks should be enhanced to recognize and evaluate the exposure risk that nanopesticides could pose to human health and the environment.

## Funders and Grant IDs

This study did not receive any fund.

## CRediT authorship contribution statement

**Jones Ackson Kapeleka:** Writing – review & editing, Writing – original draft, Project administration, Methodology, Formal analysis, Data curation, Conceptualization. **Mwema Felix Mwema:** Visualization, Investigation, Data curation.

## Data and code availability statement

Data included in article/supplementary material is referenced in the article.

## Declaration of competing interest

The authors declare that they have no known competing financial interests or personal relationships that could have appeared to influence the work reported in this paper.
